# Beneficial Effects of IABP in Anterior Myocardial Infarction Complicated by Cardiogenic Shock

**DOI:** 10.3390/medicina59101806

**Published:** 2023-10-11

**Authors:** Alberto Somaschini, Stefano Cornara, Sergio Leonardi, Andrea Demarchi, Alessandro Mandurino-Mirizzi, Federico Fortuni, Marco Ferlini, Gabriele Crimi, Rita Camporotondo, Massimiliano Gnecchi, Luigi Oltrona Visconti, Stefano De Servi, Gaetano Maria De Ferrari

**Affiliations:** 1 Cardiac Intensive Care Unit, Division of Cardiology, San Paolo Hospital, 17100 Savona, Italy; stefano.cornara@gmail.com; 2 Department of Molecular Medicine, Unit of Cardiology, University of Pavia, 27100 Pavia, Italydrandreademarchi1989@gmail.com (A.D.);; 3 Division of Cardiology, Fondazione IRCCS Policlinico San Matteo, 27100 Pavia, Italy; marco.ferlini@gmail.com (M.F.); r.camporotondo@smatteo.pv.it (R.C.); l.oltronavisconti@smatteo.pv.it (L.O.V.); 4 Cardiocentro Ticino Institute, Ente Ospedaliero Cantonale, 6900 Lugano, Switzerland; 5 Division of Cardiology, “V. Fazzi” Hospital, 73100 Lecce, Italy; 6 Interventional Cardiology Unit, CardioThoraco Vascular Department (DICATOV), IRCCS Ospedale Policlinico San Martino, 16132 Genova, Italy; gabrielecrimi@gmail.com; 7 Cardiolgia Traslazionale, Fondazione IRCCS Policlinico San Matteo, 27100 Pavia, Italy; 8 Division of Cardiology, Cardiovascular and Thoracic Department, Città della Salute e della Scienza Hospital, 10126 Turin, Italy; 9 Department of Medical Sciences, University of Turin, 10126 Turin, Italy

**Keywords:** acute myocardial infarction, cardiogenic shock, mechanical circulatory support, IABP

## Abstract

*Background and Objectives*. Recent guidelines have downgraded the routine use of the intra-aortic balloon pump (IABP) in patients with cardiogenic shock (CS) due to ST-elevation myocardial infarction (STEMI). Despite this, its use in clinical practice remains high. The aim of this study was to evaluate the prognostic impact of the IABP in patients with STEMI complicated by CS undergoing primary PCI (pPCI), focusing on patients with anterior MI in whom a major benefit has been previously hypothesized. *Materials and Methods.* We enrolled 2958 consecutive patients undergoing pPCI for STEMI in our department from 2005 to 2018. Propensity score matching and mortality analysis were performed. *Results*. CS occurred in 246 patients (8.3%); among these patients, 145 (60%) had anterior AMI. In the propensity-matched analysis, the use of the IABP was associated with a lower 30-day mortality (39.3% vs. 60.9%, *p* = 0.032) in the subgroup of patients with anterior STEMI. Conversely, in the whole group of CS patients and in the subgroup of patients with non-anterior STEMI, IABP use did not have a significant impact on mortality. *Conclusions*. The use of the IABP in cases of STEMI complicated by CS was found to improve survival in patients with anterior infarction. Prospective studies are needed before abandoning or markedly limiting the use of the IABP in this clinical setting.

## 1. Introduction

During the last decades, the survival of patients affected by ST-segment elevation myocardial infarction (STEMI) showed a dramatic increase, mainly due to improvements in evidence-based therapies including early revascularization with primary percutaneous coronary intervention (pPCI) [1,2]. Nevertheless, the occurrence of cardiogenic shock (CS) related to STEMI remains one of the major causes of death, with a growing incidence in recent years and with mortality rates approaching 40–50% [3,4,5,6,7]. In this challenging scenario, mechanical circulatory support (MCS) devices are one of the available therapeutic options to improve hemodynamics and prognosis, limiting the toxicity of catecholamines [1,6,8,9]. The intra-aortic balloon pump (IABP) has been used for more than 50 years for its documented beneficial hemodynamic effects. Specifically, the IABP increases diastolic blood pressure and coronary perfusion, while it decreases the afterload and myocardial oxygen consumption [9,10]. Despite these theoretical benefits, in the last decade, growing evidence has challenged the beneficial role of the IAP [11,12,13,14] leading to its routine use in patients with myocardial infarction (MI) complicated by CS to be qualified as a Class III (level of evidence A) recommendation in the latest European guidelines [1]. Nevertheless, the IABP still represents the most widely used MCS device in clinical practice as it is perceived by many physicians as safe, affordable, easy to use and beneficial [8,9]. Moreover, recent studies have shown a favorable effect of IABP use in some high-risk subsets of patients, including those with anterior STEMI and persistent ischemia after pPCI [15,16,17,18,19].

The aim of the present study was to evaluate the potential benefit of IABP use in a large real-world cohort of consecutive patients with STEMI complicated by CS, focusing on patients with anterior localization of the infarction.

## 2. Materials and Methods

Study population and procedures. We retrospectively analyzed our registry in which all consecutive STEMI patients who underwent pPCI at Policlinico San Matteo in Pavia, Italy, between 1 January 2005 and 30 June 2018 were prospectively enrolled. STEMI was defined according to the current guidelines at the time of patient enrollment. The current STEMI definition recognizes the presence of typical symptoms of myocardial ischemia plus either ≥1 mV ST segment elevation for ≥20 min in ≥2 contiguous electrocardiogram leads or new left-bundle branch block in the presence of modified Sgarbossa criteria or hemodynamic instability [1]. We excluded patients undergoing PCI beyond 12 h from symptoms onset (24 h in case of cardiogenic shock), rescue PCI and urgent cardiac surgery. All revascularization procedures were performed by an experienced 24 h on-call team.

Clinical data collection. For each patient, informed written consent was obtained, and the study protocol conforms to the ethical guidelines of the 1975 Declaration of Helsinki as reflected in a priori approval by the institution’s human research committee (date of Institutional Review Board approval: 1 January 2005). Demographic, clinical, procedural, electrocardiographic and laboratory data were prospectively collected into a dedicated database. Laboratory data were gathered at admittance (pre pPCI) and the following days in the Cardiac Intensive Care Unit (CICU). Detailed angiographic and procedural information of the pPCI were also collected. Follow-up data regarding the primary endpoint of this investigation (30-day all-cause mortality) were collected through an outpatient clinical visit at 30 days or via telephone contact by trained medical staff. PCI technique and all peri-procedural therapies were given according to institutional protocols and current guideline recommendations by the interventional and/or CICU cardiologists.

Definitions. The presence of a persistent (>30 min) systolic blood pressure (SBP) < 90 mmHg (or the need for pharmacological support to maintain SBP > 90 mmHg) due to cardiac dysfunction and signs of pulmonary congestion or impaired end-organ perfusion qualified for CS, in accordance with the previous literature [12]. Contrast-induced acute kidney injury (CI-AKI 0.5) was defined as a rise >0.5 mg/mL in serum creatinine occurring in the 96 h following pPCI [20]. Bleeding was defined according to TIMI criteria [21]; for the purpose of the current analysis, we considered all TIMI bleeding events, including both major or minor bleeding. According to the previous literature, ST resolution was defined as a ≥70% resolution of initial ST shift measured 20 milliseconds after the end of the QRS complex in the lead with maximal ST deviation twelve-lead ECGs performed at baseline (before coronary angiography) and at 60 min after reperfusion (elevation or depression) [22,23,24].

Statistical analysis. Categorical data were reported as absolute values and percentages. To evaluate the association between categorical data and IABP, Pearson’s χ2 test or Fisher exact test were used, as appropriate. Continuous variables were presented as median (with Q1–Q3 percentiles) and compared using Mann–Whitney U test. To evaluate the prognostic impact of IABP use, we performed propensity-matched analysis aiming to limit the influence of measured confounders. A propensity score was calculated for each patient using a logistic regression model in a dedicated analysis; the propensity score was an estimate of the probability that each patient received IABP and was created as follows. First, univariable associations were calculated for all variables known before IABP insertion that could have influenced the choice of inserting IABP. Second, all variables with *p* ≤ 0.05 (anterior STEMI, age > 75 years, three-vessel disease, persistent blood pressure ≤ 90 mmHg at admission in the cath-lab, hyperglycemia at admission, anemia at admission, TIMI final < 3, out of hospital cardiac arrest, diabetes, and female sex) were included in the propensity model. Based on the propensity score, each patient in whom IABP was used was matched to a unique control patient in whom IABP was not used. One-to-one matching was performed with nearest neighbor matching algorithm; the caliper width was equal to 0.1 of the standard deviation of the logit of the propensity score. Mortality analyses in the propensity-matched populations were performed using Kaplan–Meyer curves and log-rank test. The software used for the analysis was SPSS version 22 (SPSS Inc., Armonk, NY, USA), and the cut-off adopted for statistical significance was two-sided *p* value < 0.05.

## 3. Results

Primary PCI for STEMI was performed in 2958 patients between 1 January 2005 and 30 June 2018. Demographic, clinical, procedural and laboratory baseline variables of the overall STEMI population and the subset of patients with STEMI complicated by CS are shown in Table 1.

### 3.1. Characteristics of Patients with Cardiogenic Shock

CS occurred in 246 patients (8.3%) out of the whole population, 54.7% (*n* = 133) of whom received the IABP; 30-day mortality in CS patients was 45.5%. Among patients with STEMI complicated by CS, anterior localization of the infarction occurred in 59.8% of cases, with a higher 30-day mortality compared to patients with CS and non-anterior STEMI (49.6% vs. 37.5%, *p* < 0.001). Main significant differences in characteristics of patients with CS stratified for the localization of the infarction are shown in Table 2. Overall, patients with anterior STEMI presented a higher profile risk compared to their counterparts. The IABP was used, respectively, in 60.8% (*n* = 87) and 47.4% (*n* = 46) of patients with anterior vs. non-anterior STEMI complicated by CS (*p* = 0.04). Characteristics of patients with anterior STEMI complicated by CS stratified for IABP use are summarized in Table 3. IABP use in CS patients was neither associated with major complications nor with a significant increased rate of bleeding during hospital stay. Nevertheless, numerically greater, albeit not statistically significant, increases in the need for vascular surgery (8.5% vs. 5.5%, *p* = 0.366) and in the rate of bleeding (38.7% vs. 26.7%, *p* = 0.071) were found.

### 3.2. Mortality Analysis

In the univariate analysis for the whole CS group, IABP use was associated with a lower 30-day mortality (38.7% vs. 53%, OR 0.56, 95%CI 0.32–0.96, *p* = 0.002). In the subset of patients with anterior STEMI, IABP use was associated with a lower 30-day mortality (41% vs. 60.8%, OR 0.45, 95%CI 0.21–0.92, *p* = 0.013) whereas in the subgroup of patients with non-anterior STEMI, IABP use was not significantly associated with a lower 30-day mortality (34.1% vs. 41.3%, OR 0.74, 95%CI 0.39–1.76, *p* = 0.432).

**Figure 1:**
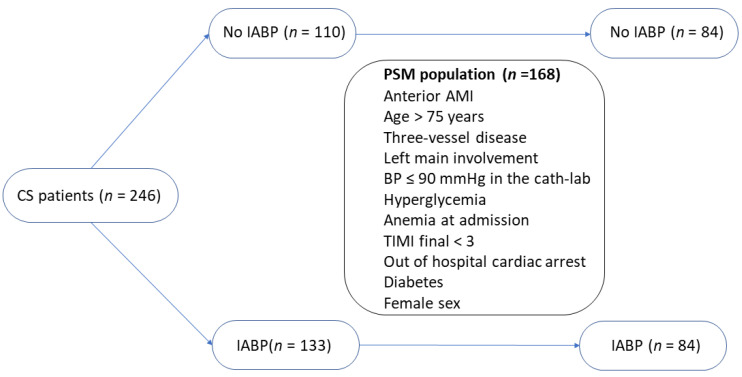
Matching flow diagram. Legend: CS = cardiogenic shock; IABP = intra-aortic balloon pump; PSM = propensity score matching; AMI = acute myocardial infarction; and TIMI = thrombolysis in myocardial infarction.

Furthermore, among 246 patients with CS, we successfully matched 84 patients who received the IABP with 84 patients who did not receive the IABP, but who showed a similar propension to receive the device according to the variables available before the decision. The matching flow diagram is presented in Figure 1. Characteristics of the two groups are summarized in Table 4.

**Figure 2:**
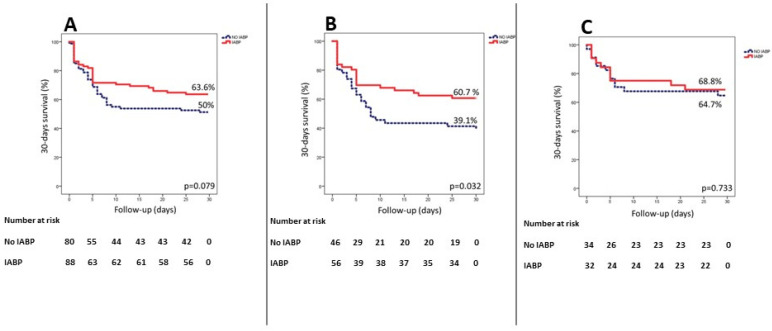
Panel (**A**): cumulative incidence of 30-day survival in the overall matched population of patients with STEMI complicated by cardiogenic shock; panel (**B**): cumulative incidence of 30-day survival in the subset of patients with anterior STEMI; and panel (**C**): cumulative incidence of 30-day survival in the subset of patients with non-anterior STEMI.

Figure 2 illustrates the Kaplan–Meier curves for 30-day mortality in the matched population for patients who received the IABP vs. those who did not receive the IABP in the whole group of CS patients (panel A) and in the subsets of patients with anterior (panel B) and non-anterior (panel C) STEMI. Indeed, in the first group, the overall 30-day mortality was 42.9% (*n* = 72), specifically 36.4% (*n* = 32) in patients in whom the IABP was used and 50% (*n* = 40) in the others (*p* = 0.079). In the subset of patients with anterior STEMI, the overall 30-day mortality was 49% (*n* = 50) to 39.3% (*n* = 22) in patients who received the IABP and 60.9% (*n* = 28) in those who did not (*p* = 0.032). Finally, in patients with non-anterior myocardial infarction, there was no significant difference in 30-day mortality according to IABP use (34.2% vs. 35.3%, *p* = 0.733).

## 4. Discussion

The main finding of the present study is that treatment with IABP in patients with anterior STEMI complicated by cardiogenic shock is associated with decreased short-term mortality.

Cardiogenic shock due to STEMI still has an unacceptable mortality rate of 40–50% [3,4,5,6,7]. MCS devices represent an interesting therapeutic opportunity, as they offer the chance to assist the failing cardiac pump, limiting the use of intravenous inotropes, which may worsen myocardial ischemia. In our cohort of nearly three-thousands STEMI patients, 8.3% presented with CS with a short-term mortality of 45.5%, in accordance with the previous literature [3,4,5,6,7]. The IABP is still the most used device in patients with STEMI complicated by CS [8,9]. Despite this, its routine use has been downgraded to a class III recommendation [1]. The widespread use of the IABP can be explained considering several advantageous features: simplicity of use, an elevated safety profile, a low cost and a high perception of its usefulness by the operators [8,9,13,19]. Moreover, robust evidence in favor of more complex devices (i.e., axial pumps or percutaneous LVADs) in this clinical context is still lacking [25,26,27,28,29].

The downgrading of IABP routine use in the guidelines was mostly ascribable to the data provided by the IABP-SHOCK II trial. This was the first and only adequately powered randomized clinical trial evaluating the prognostic impact of the IABP in CS due to myocardial infarction, and it documented a neutral effect on short-term mortality, mainly due, according to the authors, to the modest effects of the device on cardiac output [12]. Long-term follow-up data at 12 months and 6 years were consistent with the initial findings of the study [30,31]. Despite its undoubted value as a landmark study in the field, a number of criticisms have been raised, in particular for the presence of a high rate of non-ST elevation MI (roughly 1/3 of the total population) and non-anterior STEMI patients (nearly 50%), who are less likely to receive a benefit from the IABP. Moreover, the lower than expected incidence of events in the control group configured the trial to be underpowered with regard to the primary hypothesis. Therefore, given the heterogeneous population enrolled in the trial, it is possible that a potential benefit of the IABP was lost, which could be present in peculiar high-risk subsets of patients such as those with an anterior STEMI. According to this hypothesis, some recent studies have found a potential benefit of non-routine IABP use in high-risk STEMI subgroups [16,17,18].

Anterior localization of myocardial infarction is a well-known marker of high risk. Indeed, among our STEMI patients, it was associated with a higher mortality (7.1% vs. 3.7%, *p* < 0.001), higher CK peak and inflammatory markers, a lower LVEF and higher rate of hyperglycemia, incomplete ST resolution, TIMI flow < 3 after revascularization, CI-AKI and bleeding complications. Due to all these features, patients with anterior MI appear to be a good target for interventions aiming to support systemic perfusion without increasing myocardial oxygen consumption. Accordingly, the CRISP-AMI trial [19] explored the potential benefit of the routine use of the IABP in anterior MI not complicated by CS. Despite the trial being neutral, a subsequent sub-study showed a prognostic benefit in patients with larger infarction (defined as total sum of ST elevation > 15 mm) or incomplete ST resolution after reperfusion [18]. In our population, the IABP was used more frequently in patients with CS and anterior STEMI. This group of patients presented the greatest risk profile features as illustrated in Table 3, showing lower LVEF, greater CK peak and white blood cell values at admission and a numerically higher incidence of out-of-hospital cardiac arrest. Interestingly, patients with advanced peripheral artery disease were less likely to receive the IABP, possibly due to the fear of technical difficulties in the insertion. Of note, the median age was higher in patients who did not receive the IABP; nevertheless, elderly age was included in the propensity score and thus its influence on the mortality analysis is unlikely. The IABP was associated with a survival benefit in the overall population in the univariate analysis. However, after stratification for infarct localization, the benefit was consistent only in patients with anterior MI. Nonetheless, these results could be the consequence of a selection bias (i.e., the device utilization preferably in those patients where is not perceived to be futile). To account for known confounding factors, we performed a propensity matching score including in the match only the variables known before the insertion of the IABP, as they could influence the choice of inserting the device but could not be influenced by the device itself. With this method, we obtained two propensity-matched cohorts of patients each made of 84 patients with a similar propension to receive the IABP. As this constitutes a retrospective analysis, we opted not to provide a formal sample size calculation. However, it is important to acknowledge that the matching process did result in a reduction in the population of patients with CS included in the analysis, potentially affecting the power of our analysis. In the overall CS population, there was a non-significant trend towards a better survival in patients in whom the IABP was used. In the subset of patients with anterior infarction and CS, the benefit of IABP use was shown to be significant (39.3% vs. 60.9%, *p* = 0.032), while this beneficial effect was not present in those with non-anterior STEMI.

The greater benefit of the IABP in patients with CS and anterior STEMI could be explained considering several factors. First, the larger infarct size (expressed by a significantly higher CK peak) in these patients suggests a greater portion of jeopardized but potentially salvageable myocardium. The balloon pump, offering both systolic unloading and diastolic coronary flow augmentation, could help to increase oxygen delivery and decrease its consumption. In fact, the ratio between these two factors is a key factor in the ischemic damage setting [32]. With regard to this, the possible benefit of pre-revascularization 30 min unloading of the left ventricle using Impella CP in patients with STEMI without cardiogenic shock was investigated in the Door-To-Unload in STEMI pilot trial [33]. The trial was neutral regarding major adverse cardiovascular and cerebrovascular events and the infarct size measured at 30 days, demonstrating the feasibility and safety of this strategy, which is currently under investigation in a larger and adequately powered study (NCT03947619).

Second, as demonstrated by De Silva et al. [34], the effect of the IABP in augmenting the diastolic coronary perfusion is counterbalanced by the vasoconstriction of coronary arteries due to the self-regulation of the coronary circle. Thus, only when these self-regulatory mechanisms are lost (i.e., in the context of persistent ischemia as suggested by the authors), was a beneficial increase in the coronary flow due to the IABP observed. This condition is more likely to happen in the context of CS due to anterior infarction, as suggested by a significantly lower rate of ST resolution (61% vs. 78%, *p* < 0.001).

Finally, a certain number of patients with CS in the context of inferior MI are more likely to experience hypotensive episodes due to marked vagal activation or acute right ventricular dysfunction rather than true states of CS. In such cases, a prompt revascularization on top of OMT can reverse the hypotension, whereas MCS devices would not be beneficial.

### Limitations

The present analysis has some limitations. First, our study was a single-center study. Thus, our results refer to a specific population, so they could be potentially confounded by local practice and may not apply to different populations around the world. Second, we did not assess the effects of the IABP in a randomized study, but with a post hoc analysis of consecutive patients using propensity score matching to account for imbalances between groups. Therefore, it cannot be excluded that some confounders not considered in the matching process could be unevenly distributed between the two groups in this way, affecting the results of the study. Third, we did not systematically collect data regarding serum lactates, the length of IABP use and the timing of IABP insertion (i.e., before or after the procedure); nevertheless, this last variable did not have an impact on prognosis in several studies [35,36]. Fourth, at the time of data gathering we did not use other percutaneous MCS devices such as Impella in our center, thus we could not make comparisons between patients treated with different MCS devices.

## 5. Conclusions

The present analysis shows that the use of the IABP in patients with STEMI and CS is associated with a reduced short-term mortality in patients with anterior infarction, whereas it has no effect on the outcome in patients presenting with non-anterior infarction. Future prospective randomized trials aiming to evaluate the prognostic role of IABP use in patients with CS due to myocardial infarction should focus on this specific high-risk subset of patients.

## Figures and Tables

**Table 1:** Characteristics of the overall population of the study and of the subset of patients with STEMI complicated by cardiogenic shock.

Demographic Variables	STEMI (*n* = 2959)	STEMI + Cardiogenic Shock (*n* = 246)	*p* Value
Age (years; median (Q1–Q3))	63 (54–73)	69.5 (60–77)	<0.001
Age > 75 years (*n*, %)	613 (20.4%)	83 (33.7%)	<0.001
Female sex (*n*, %)	663 (22%)	75 (30.5%)	<0.001
Body mass index (kg/m^2^; median (Q1–Q3))	25.8 (23.4–28.7)	24.2 (22.8–26.2)	<0.001
CV risk factors			
Smoke (*n*, %)	1861 (63.1%)	94 (38.5%)	<0.001
Hypertension (*n*, %)	1631 (55.3%)	46 (60.1%)	<0.001
Type II diabetes mellitus (*n*, %)	496 (16.8%)	46 (18.9%)	<0.001
Dyslipidemia (*n*, %)	1162 (39%)	55 (22.6%)	<0.001
Family history of cardiovascular disease (*n*, %)	966 (32.8%)	39 (16%)	<0.001
Medical history			
Peripheral arterial disease (*n*, %)	312 (10.4%)	47 (19.4%)	<0.001
Previous myocardial infarction (*n*, %)	392 (13.3%)	40 (16.4%)	0.056
Previous percutaneous coronary intervention (*n*, %)	357 (12.1%)	33 (13.5%)	0.199
Previous coronary artery bypass grafting (*n,* %)	53 (1.8%)	8 (3.3%)	0.712
Chronic kidney disease (*n*, %)	642 (21.4%)	105 (52.8%)	<0.001
Clinical and laboratory variables			
Out of hospital cardiac arrest (*n*, %)	284 (9.8%)	92 (37.9%)	<0.001
Anemia (*n*, %)	528 (8.1%)	106 (45.5%)	<0.001
Heart rate (bpm; median (Q1–Q3))	75 (65–87)	80 (67–99)	0.001
Left ventricular ejection fraction (median(Q1–Q3))	45 (38–50)	35 (25–40)	<0.001
Systolic arterial pressure (mmHg; median (Q1–Q3))	135 (120–150)	90 (75–103)	<0.001
Baseline blood sugar (mg/dL; median (Q1–Q3))	141 (120–176)	175 (136–219)	<0.001
Hyperglycemia (*n*,%)	469 (16.5%)	95 (43%)	<0.001
Baseline hemoglobin (g/dL; median (Q1–Q3))	14.3 (13.5–15.3)	13.6 (12–14.7)	<0.001
Baseline white blood cells (n × 10^3^/mcl; median (Q1–Q3))	11.2 (8.8–13.7)	13.3 (9.5–20)	<0.001
Troponin I peak (ng/dL; median (Q1–Q3))	80 (32–166)	196 (81–355)	0.001
Creatine kinase peak (MU/L; median (Q1–Q3))	1.3 (0.6–2.4)	2.8 (1.3–5.6)	<0.001
Baseline creatinine (mg/dL; median (Q1–Q3))	0.9 (0.8–1.1)	1 (0.8–1.3)	<0.001
ECG			
Anterior STEMI (*n*, %)	1240 (46.5%)	145 (59.7%)	<0.001
ST resolution (*n*, %)	1908 (69.9%)	98 (49%)	<0.001
Procedural data			
Three-vessel disease (*n*, %)	772 (26.4%)	86 (36.3%)	0.001
Left main involvement (*n*, %)	32 (1.1%)	18 (8.1%)	<0.001
Blood pressure ≤ 90 mmHg in the cath-lab (*n*,%)	90 (3.1%)	46 (20.3%)	<0.001
Post-procedural TIMI flow <3 (*n*, %)	278 (9.3%)	56 (23.5%)	<0.001
Length of stay			
Coronary care unit (days; median (Q1–Q3))	4 (3–5)	6 (2–10)	<0.001
Hospital (days; median (Q1–Q3))	7 (6–9)	9 (4–14)	<0.001

**Table 2:** Characteristics of patients with STEMI complicated by CS in patients with anterior vs. non anterior myocardial infarction.

Variables	Anterior STEMI		*p*-Value
	NO (*n* = 101)	YES (*n* = 145)	
Age > 75 years (*n,* %)	39 (39.8%)	44 (30.1%)	0.128
Female sex (*n*, %)	34 (34.7%)	39 (26.9%)	0.193
Type II diabetes mellitus (*n*, %)	21 (21.9%)	25 (17.4%)	0.348
Heart rate (bpm; median (Q1–Q3))	73 (62–82)	80 (68–88)	<0.001
Anemia (*n*, %)	43 (46.7%)	61 (44.2%)	0.075
Out of hospital cardiac arrest (*n*, %)	29 (30.2%)	61 (42.4%)	0.057
Hyperglycemia (*n*, %)	34 (39.5%)	59 (44.7%)	0.451
Creatine kinase peak (Mu/L; median (Q1–Q3))	1.2 (0.6–2.1)	1.7 (0.8–3)	<0.001
Baseline white blood cells (n × 10^3^/mcl; median (Q1–Q3))	10.8 (8.7–13.1)	11.5 (9–14.2)	<0.001
Left ventricular ejection fraction (%; median(Q1–Q3))	40 (30–47)	27 (20–37.5)	<0.001
Contrast-induced acute kidney injury (*n*, %)	5 (5.4%)	13 (9.3%)	<0.001
Bleeding (*n*, %)	9 (9.6%)	22 (15.6%)	<0.001
Three-vessel disease (*n*, %)	33 (34.7%)	53 (38.1%)	0.597
Left main involvement (*n*, %)	1(1.2%)	16 (11.9%)	0.004
Systolic blood pressure < 90 mmHg in the cath-lab (*n*, %)	23 (25%)	22 (16.7%)	0.126
Post-procedural TIMI flow <3 (*n*, %)	23 (23.7%)	32 (23.2%)	0.926
ST resolution (*n*, %)	78 (78%)	88 (61%)	<0.001

**Table 3:** Characteristics of patients with anterior AMI complicated by CS stratified for IABP use.

Variables	IABP		*p*-Value
	NO (*n* = 58)	YES (*n* = 87)	
Age (years; median (Q1–Q3))	74 (68–82)	67 (58–74)	<0.001
Age > 75 years (*n*, %)	22 (39.3%)	21 (24.1%)	0.054
Body mass index (kg/m^2^, median (Q1–Q3))	24.2 (22.8–26.9)	24.5 (22.8–28.7)	0.535
Female sex (%)	16 (28.6%)	21 (24.1%)	0.555
Hypertension (%)	35 (62.5%)	44 (51.2%)	0.184
Type II diabetes mellitus (%)	7 (12.5%)	18 (20.9%)	0.197
Anemia (*n*, %)	29 (55.8%)	32 (37.6%)	0.038
Previous myocardial infarction (%)	12 (21.4%)	13 (14.9%)	0.319
Peripheral arterial disease (%)	16 (27.8%)	12 (14%)	0.044
Out of hospital cardiac arrest (%)	19 (34.5%)	40 (46.5%)	0.178
Heart rate (bpm; median (Q1–Q3))	80 (72–102)	91 (69–109)	0.706
Baseline blood sugar (mg/dL; median (Q1–Q3))	141 (133–181)	214 (176–263)	0.005
Left ventricular ejection fraction (median(Q1–Q3))	30 (25–40)	25 (20–35)	0.041
Baseline hemoglobin (g/dL; median (Q1–Q3))	12.3 (11.4–15.1)	13.9 (12.4–15.1)	0.058
Baseline platelets (Mu/L, median (Q1–Q3))	232 (206–266)	246 (203–304)	0.276
Platelets nadir (Mu/L, median (Q1–Q3))	188 (159–228)	137 (101–200)	<0.001
Baseline white blood cells (n × 10^3^/mcl; median (Q1–Q3))	12.49 (9.8–16.4)	14.6 (11.4–21.3)	0.013
Hyperglycemia (*n*, %)	16 (31.4%)	42 (52.5%)	0.018
Creatine kinase peak (mg/dMU/L; median (Q1–Q3))	2.83 (1.04–4.34)	4.06 (2.7–7.1)	0.001
Baseline eGFR * (mg/mL, median (Q1–Q3))	44 (36–74)	65 (43–88)	0.087
Systolic blood pressure < 90 mmHg in the cath-lab (*n*, %)	3 (5.4%)	19 (25%)	0.003
Three-vessel disease (%)	22 (40%)	31 (37.3%)	0.754
Left main involvement (*n*, %)	2 (3.7%)	12 (15.2%)	0.034
ST resolution (%)	17 (34.7%)	32 (50%)	0.104
CI-AKI 0.5 (%)	14 (26.4%)	21 (25%)	0.853
Post-procedural TIMI flow <3 (%)	13 (23.6%)	18 (22%)	0.817
GP IIbIIIa—inhibitors use (%)	19 (37.3%)	43 (52.4%)	0.088
Days in Coronary Care Unit (days; median (Q1–Q3))	7 (4–10)	6 (4–10)	0.219
Days in hospital (days; median (Q1–Q3))	8 (6–15)	12 (8–19)	0.003

Legend: eGFR = estimated glomerular filtration rate; CI-AKI = contrast-induced acute kidney injury. * eGFR was estimated using Cockroft–Gault formula.

**Table 4:** Characteristics of propensity-matched populations stratified for IABP use.

Variables Included in the PMS	IABP		*p*-Value
	NO (*n* = 84)	YES (*n* = 84)	
Age > 75 years (%)	30 (37.5%)	26 (29.5%)	0.275
Anterior myocardial infarction (%)	46 (57.5%)	56 (63.6%)	0.416
Three-vessel disease (%)	26 (32.5%)	32 (36.4%)	0.599
Left main involvement (%)	2 (2.5%)	6 (6.8%)	0.189
Systolic blood pressure < 90 mmHg in the cath-lab (*n*, %)	60 (62.5%)	66 (75%)	0.080
Hyperglycemia at admission (%)	26 (32.5%)	44 (50%)	0.022
Anemia at admission (%)	41 (51.2%)	34 (38.6%)	0.100
Post-procedural TIMI flow <3 (%)	17 (21.3%)	20 (22.7%)	0.817
Out of hospital cardiac arrest (%)	23 (28.7%)	18 (20.5%)	0.211
Type II diabetes mellitus (%)	11 (13.8%)	22 (25%)	0.067
Female sex (%)	24 (30%)	27 (30.7%)	0.924

## Data Availability

The data presented in this study are available on request from the corresponding author.
